# Medicare Savings Program Take-Up Estimates and Profile of Enrolled and Unenrolled Individuals

**DOI:** 10.1001/jamanetworkopen.2025.35408

**Published:** 2025-10-03

**Authors:** Sarah Kotb, Amanda Su, Anna D. Sinaiko

**Affiliations:** 1 Economics Department, College of Social and Behavioral Science, University of Utah, Salt Lake City; 2 Department of Health Policy, Stanford University, Stanford, California; 3 Department of Health Policy and Management, Harvard TH Chan School of Public Health, Boston, Massachusetts

## Abstract

**Importance:**

Medicare enrollees with low income report challenges affording out-of-pocket costs for health care. Although the Medicare Savings Programs (MSPs) were established to provide financial support, recent patterns in program take-up are understudied.

**Objectives:**

To provide national and state-level estimates of take-up of the MSPs from 2018 to 2020 and describe the profile of enrolled and unenrolled individuals eligible for the MSPs.

**Design, Setting, and Participants:**

This cross-sectional analysis used data from the Medicare Current Beneficiary Survey (MCBS), a nationally representative survey of community-dwelling Medicare beneficiaries. Respondents from 2018 to 2020 who completed the income and assets questionnaire, which allowed assessment of MSP eligibility, were analyzed. Data were analyzed in July 2024.

**Exposures:**

Eligibility for the MSPs.

**Main Outcomes and Measures:**

The take-up rate of the MSPs, defined as the proportion of eligible beneficiaries enrolled in the program. The MCBS survey weights were applied to create a subsample that was nationally representative of the community-dwelling Medicare population.

**Results:**

The primary sample included 26 240 respondent-year observations, representing 179 221 355 beneficiary-years (14.0% [95% CI, 13.4%-14.5%] of respondents were <65 years, 55.1% [95% CI, 54.1%-56.0%] were female, and 37.7% [95% CI, 36.0%-39.4%] had a high school education or lower). A total of 20.9% (95% CI, 19.8%-22.0%) of the primary sample was eligible for the MSPs. Of those eligible, 56.7% (95% CI, 54.5%-59.0%) were enrolled. Take-up rates varied widely across states, ranging from 41.5% (95% CI, 25.7%-57.3%) in Ohio to 72.9% (95% CI, 67.6%-78.2%) in California. Take-up among Medicare Advantage beneficiaries was higher than among those in traditional Medicare (61.3% vs 52.9%; difference, 8.4 percentage points [pp] [95% CI, 3.5-13.2 pp]). Compared with eligible beneficiaries who were not enrolled, enrolled individuals had greater economic insecurity, including being 30.0 pp (95% CI, 25.4-34.6 pp) more likely to report income below 100% of the federal poverty level and 16.4 pp (95% CI, 13.2-19.6 pp) more likely to report assets less than $3000.

**Conclusions and Relevance:**

This cross-sectional study of Medicare beneficiaries suggests that MSP take-up remains incomplete and varied across states despite policy efforts. A policy to encourage participation in the MSPs among eligible populations that target less socially and financially vulnerable—although still with low income and eligible for the MSPs—individuals may be more likely to be associated with gains in the MSP take-up.

## Key Points

**Question:**

What are state and national estimates of take-up of the Medicare Savings Programs (MSPs), and what are the characteristics of enrolled individuals and unenrolled individuals eligible for the MSPs?

**Findings:**

In this cross-sectional study representing 179 221 355 beneficiary-years, 56.7% of those eligible for the MSPs from 2018 to 2020 were enrolled; take-up varied across states and was higher among Medicare Advantage beneficiaries compared with traditional Medicare enrollees. Enrolled individuals had worse health, lower income, and fewer assets than individuals eligible for the MSPs who did not enroll.

**Meaning:**

This study suggests that the MSPs are serving beneficiaries with greater financial and health insecurity; a policy to encourage participation among other eligible individuals is more likely to increase MSP take-up.

## Teaser

This cross-sectional study estimates national and state-level enrollment in the Medicare Savings Programs from 2018 to 2020 and describes characteristics of enrolled and unenrolled eligible individuals.

## Introduction

For individuals aged 65 years or older and individuals with qualifying disabilities, Medicare health insurance reduces the costs of medical care but requires substantial cost sharing, including premiums, deductibles, and coinsurance.^1^ This burden disproportionately affects beneficiaries with low income. In a 2022 survey, 50% of Medicare respondents with low income reported difficulty accessing health care due to cost, 39% reported difficulty affording monthly premiums, and 23% faced challenges paying medical bills or debt.^2,3^

Concerns about the burden of Medicare’s high cost sharing have existed since the 1980s. In response, the Medicare Savings Programs (MSPs) were enacted in 1988 to protect qualifying Medicare beneficiaries with low income from these financial burdens.^4,5,6^ These programs have federal eligibility standards. In 2020, for example, a Medicare beneficiary in a single-person household was eligible if they had a monthly income lower than $1400 and owned countable assets valued less than $12 000. In addition, 13 states and the District of Columbia instated more generous criteria for their residents; 2 states raised their income limit, 7 states increased or eliminated the asset limit, and 4 states and Washington, DC, applied both approaches.^7^

Once enrolled in the MSPs, beneficiaries receive premium and/or cost-sharing assistance from their state’s Medicaid agency, with estimates indicating out-of-pocket spending reductions of up to $1144 per year.^8^ The scope of assistance varies by MSP: the Qualified Medicare Beneficiary (QMB) program covers both Medicare Parts A and B premiums and cost sharing, while the Specified Low-Income Medicare Beneficiary (SLMB) and Qualifying Individual (QI) programs cover the Part B premium only.^9^ Although not all MSP enrollees qualify for full Medicaid benefits, some do, and for those beneficiaries, Medicaid may also cover additional services, such as dental, long-term care, and nonemergency medical transportation. Moreover, MSP enrollment automatically qualifies individuals for the Part D Low-Income Subsidy (LIS), which helps cover prescription drug premiums and cost sharing, further extending a beneficiary’s financial protection.^10^ Regardless of full Medicaid or LIS eligibility, the core value of the MSPs lies in their ability to reduce out-of-pocket costs associated with Medicare Parts A and B. These savings can potentially reduce the need for purchasing supplementary insurance via Medigap or enrolling in a private Medicare plan (ie, Medicare Advantage) in which enrollees have reduced cost sharing but stricter provider networks.^11,12^

After evidence of low take-up of MSPs emerged in the 2000s—only one-third to one-half of eligible individuals were enrolled^5,13,14,15,16^—several policy efforts aimed to improve take-up. For example, the Medicare Improvements for Patients and Providers Act of 2008 relaxed asset eligibility requirements and increased outreach to eligible individuals.^17,18^ Although these efforts have been credited with streamlining enrollment into the MSPs, a comprehensive understanding of current MSP take-up remains limited.^18^ One recent study estimated that, depending on the specific MSP, between 24% and 83% of eligible beneficiaries were unenrolled from 2018 to 2021.^19^ However, the variation in take-up across states and by type of Medicare insurance (ie, Medicare Advantage [MA] vs traditional Medicare [TM]) remains poorly understood. The characteristics of unenrolled beneficiaries—insights that are needed to guide targeted take-up policy—are also not well understood. In this study, we use 2018 to 2020 data to provide new estimates of how MSP take-up varies across these dimensions and to describe the profile of enrolled and unenrolled individuals among the population eligible for the MSPs.

## Methods

The Harvard Medical School institutional review board has approved this study and granted a waiver for informed consent because the study relied on secondary data and presented minimal risk to participants; in addition, obtaining individual informed consent would not be feasible. We followed the Strengthening the Reporting of Observational Studies in Epidemiology (STROBE) reporting guideline for cross-sectional studies.

### Data Sources and Study Population

We conducted a cross-sectional analysis using the 2018 to 2020 Medicare Current Beneficiary Survey (MCBS), an annual, nationally representative survey of Medicare beneficiaries. The MCBS reports income and financial assets as well as state of residence and marital status, all of which are used to measure MSP eligibility. We matched each respondent with their state’s MSP income and asset limits for single and married beneficiaries, obtained from the National Council on Aging (see eAppendix 1 in Supplement 1 for a summary of these rules).^7,20,21^ We restricted our analysis to respondent-years in which individuals completed the detailed income and asset questionnaire, and we applied the MCBS survey weights so that this subsample is nationally representative of the community-dwelling Medicare population (see eAppendix 2 in Supplement 1 for details on sample construction and representativeness). We included both full-benefit dually eligible individuals, who receive full Medicaid benefits in addition to MSP assistance, and partial-benefit dually eligible individuals, who receive MSP assistance only. We linked this sample to administrative enrollment records from the Centers for Medicare & Medicaid Services (CMS), which report individual-level MSP enrollment.

### Measures of MSP Eligibility, Enrollment, and Take-Up

We constructed a measure of individual eligibility for the MSPs in 3 steps. First, we followed the MSP methodology and measured an individual’s countable income, applying the program’s deductions (eg, half of wages are not included). Second, we used MSP guidelines to calculate countable assets as the sum of the value of liquid cash and illiquid assets (eg, property and stocks), again applying MSP program deductions (eg, primary residence is not included). Finally, we compared each individual’s income and assets against MSP rules specific to their state and marital status to determine their eligibility. In a sensitivity analysis, we assessed eligibility rates using alternative definitions of income and assets (see eAppendix 3 in Supplement 1 for these measures and their validation).

We measured individual-level MSP enrollment directly from the CMS administrative enrollment data. We constructed national and state-level measures of take-up of the MSPs, which we defined as the share of population estimated to be eligible that was enrolled in the program. Our primary measures examined eligibility and take-up averaged across all 3 major MSP subprograms (QMB, SLMB, and QI).

### Covariates

We used data from the MCBS to assess beneficiaries’ demographic characteristics, economic characteristics, and health profiles. Demographic characteristics included age younger than 65 years (indicating Medicare eligibility based on disability), marital status, educational attainment, sex, and English proficiency. Race and ethnicity were based on the respondents’ self-identification, with individuals able to choose more than 1 race (American Indian or Alaska Native, Asian, Black or African American, Native Hawaiian or Pacific Islander, and White), and this information was combined with their reported Hispanic ethnicity. Race and ethnicity were included because MSP enrollees and unenrolled individuals eligible for the MSPs may differ across racial and ethnic characteristics, and documenting these differences helps characterize variation in program take-up.

Economic characteristics were individual’s and (if applicable) spousal income and assets. We also included indicators for whether the household was in an economically deprived location based on the Area Deprivation Index, in an urban area, and whether the household reported food insecurity.

Our 5 measures of health were whether the beneficiary reported having depression, vision problems, hearing problems; difficulty performing any activities of daily living, including walking, eating, dressing, and bathing; and difficulty performing any instrumental activities of daily living, including making telephone calls, shopping, and managing money. See eAppendix 4 in Supplement 1 for further detail on these measures and the handling of missing observations.

### Statistical Analysis

Statistical analysis was performed in July 2024. We reported estimates of MSP eligibility and MSP take-up nationally, by state, and by the type of Medicare insurance (ie, TM vs MA). The state-level analysis included states with at least 100 MCBS respondents in a given year to reduce measurement error. These 26 states are representative of 92.3% of Medicare beneficiaries (ie, 51 million of the 55 million community-dwelling Medicare beneficiaries represented in the MCBS in 2018, the first year of our sample period). To estimate 95% CIs in these take-up rates, we constructed variances using the balanced repeated replication method, which accounts for the nonrandom sampling nature of the survey.^22^

To examine differences between the enrolled and unenrolled eligible populations, we separately reported the mean value of a given characteristic for each of the 2 groups.^5,14,16,23^ We also reported the difference between the 2 mean values and its 95% CI, estimated from linear regression models regressing the characteristic on enrollment status among eligible individuals. Because individuals may use their assets to finance Medicare cost sharing, we compared asset ownership across the 2 groups. We also replicated these analyses with state fixed effects to account for potential confounding with the state of residence. All statistical tests were 2-sided.

## Results

### Sample Characteristics

The primary sample included 26 240 observations reflecting 170 221 355 beneficiary-years (55.1% [95% CI, 54.1%-56.0%] were female and 44.9% [95% CI, 44.0%-45.9%] were male); 14.0% (95% CI, 13.4%-14.5%) were younger than 65 years, meaning that they received Medicare because of a qualifying disability; 37.7% (95% CI, 36.0%-39.4%) had a high school degree or lower; 14.8% (95% CI, 13.7%-15.8%) had income under 100% of the federal poverty level (FPL); and 32.8% (95% CI, 31.4%-34.1%) had countable assets under $3000 (Table 1). The sample was 1.2% (95% CI, 0.6%-1.7%) American Indian or Alaska Native and Native Hawaiian or Pacific Islander (data for these groups were merged due to small sample sizes and CMS restrictions on minimum required cell sizes for reporting), 2.8% (95% CI, 2.3%-3.3%) Asian, 11.0% (95% CI, 10.2%-11.9%) Black or African American, 8.3% (95% CI, 7.2%-9.4%) Hispanic, and 85.5% (95% CI, 84.5%-86.5%) White. Just over half the sample (53.5% [95% CI, 52.6%-54.5%]) reported having a difficulty in 1 or more instrumental activities of daily living.

**Table 1.** Primary Sample Characteristics

Characteristic	Weighted % (95% CI)
Demographic characteristics	
Age <65 y (qualified through a disability)	14.0 (13.4-14.5)
Not married	47.1 (45.9-48.3)
High school degree or lower	37.7 (36.0-39.4)
Female	55.1 (54.1-56.0)
Limited English proficiency	4.7 (4.0-5.4)
Race and ethnicity^a^	
American Indian or Alaska Native and Native Hawaiian or Pacific Islander	1.2 (0.6-1.7)
Asian	2.8 (2.3-3.3)
Black or African American	11.0 (10.2-11.9)
Hispanic	8.3 (7.2-9.4)
White	85.5 (84.5-86.5)
Economic characteristics	
Income <100% federal poverty level	14.8 (13.7-15.8)
Assets <$3000	32.8 (31.4-34.1)
Top quartile of Area Deprivation Index	19.9 (17.4-22.5)
Urban residence	81.1 (78.8-83.3)
Food insecure	12.1 (11.4-12.7)
Health and daily activities	
Moderately severe or severe depression	15.8 (15.0-16.5)
Vision problems	7.5 (7.0-8.1)
Hard of hearing	15.8 (15.0-16.6)
Difficulty in any activity of daily living	25.6 (24.9-26.4)
Difficulty in any instrumental activity of daily living	53.5 (52.6-54.5)
No. of person-years	170 221 355

^a^ Race and ethnicity are self-reported, and survey respondents could choose more than 1 race. We do not report American Indian or Alaska Native and Native Hawaiian or Pacific Islander races separately due to the small sizes and restrictions by the Centers for Medicare & Medicaid Services on minimum required cell sizes for reporting.

### MSP Eligibility and Take-Up

Among the primary sample, 35 587 360 beneficiary-years—represented by 6608 observations—were deemed eligible for the MSPs. That is, 1 in 5 beneficiary-years (20.9% [95% CI, 19.8%-22.0%]) among community-dwelling Medicare beneficiaries was eligible for the MSPs from 2018 to 2020 (Table 2). Eligible beneficiaries were more likely to have qualified for Medicare via disability, be unmarried, and have lower income, education, English proficiency, and health status than those not eligible for the MSPs (eAppendix 4 in Supplement 1).

**Table 2.** Medicare Savings Programs Eligibility and Take-Up^a^

Characteristic	Eligible, % (95% CI)	Eligible difference, percentage points (95% CI)	Take-up, % (95% CI)	Take-up difference, percentage points (95% CI)
All	20.9 (19.8 to 22.0)	NA	56.7 (54.5 to 59.0)	NA
By year				
2018	21.5 (20.1 to 23.0)	[Reference]	57.8 (54.9 to 60.7)	[Reference]
2019	21.0 (19.6 to 22.4)	−0.6 (−1.8 to 0.7)	55.7 (52.6 to 58.8)	−2.1 (−5.4 to 1.3)
2020	20.3 (19.1 to 21.4)	−1.3 (−2.7 to 0.2)	56.7 (53.9 to 59.6)	−1.0 (−4.4 to 2.3)
By Medicare type				
Traditional Medicare	18.3 (16.9 to 19.7)	[Reference]	52.9 (49.3 to 56.6)	[Reference]
Medicare Advantage	25.1 (23.3 to 26.9)	6.8 (4.5 to 9.1)	61.3 (58.4 to 64.2)	8.4 (3.5 to 13.2)

Abbreviation: NA, not applicable.

^a^ The Medicare Current Beneficiary Survey–provided survey weights are applied to obtain national eligibility and take-up rates. Take-up is calculated only among those who are categorized as eligible after applying the state-level Medicare Savings Program rules.

Among eligible beneficiary-years, 56.7% (95% CI, 54.5%-59.0%) were enrolled in the MSPs (Table 2). Results from analysis using an alternate construction of income and assets and controlling for the beneficiary’s state are similar (eAppendix 5 in Supplement 1). Take-up rates by MSP subprogram vary, ranging from more than half for the QMB program to less than 20% for the SLMB and the QI programs (eAppendix 5 in Supplement 1).

### MSP Eligibility and Take-Up by Medicare Type

There were different patterns of MSP eligibility and take-up across TM and MA enrollees. A higher share of MA enrollees was eligible for the MSPs in comparison with TM enrollees (25.1% [95% CI, 23.3%-26.9%] vs 18.3% [95% CI, 16.9%-19.7%]; difference, 6.8 percentage points [pp] [95% CI, 4.5-9.1 pp]) (Table 2). Among those who were eligible, there was a larger take-up rate in MA than in TM (61.3% [95% CI, 58.4%-64.2%] vs 52.9% [95% CI, 49.3%-56.6%]; difference, 8.4 pp [95% CI, 3.5-13.2 pp]).

### Variation in MSP Take-Up Across States

**Figure 1.**
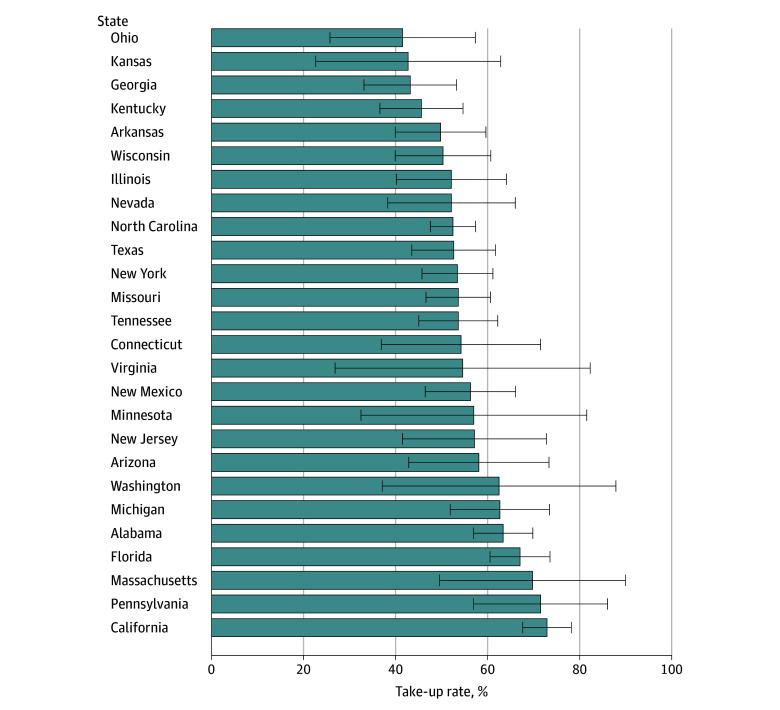
Observed Medicare Savings Programs Take-Up in 2018-2020, by State The data include 26 states with at least 100 respondents per state-year. These states represent approximately 92.3% of Medicare beneficiaries (ie, 51 million of the 55 million community-dwelling Medicare beneficiaries) in 2018, the first year of our sample period. Medicare Current Beneficiary Survey–provided national probability weights are used. Error bars indicate 95% CIs.

Medicare Savings Program take-up among eligible beneficiaries varied across states, ranging from a low of 41.5% (95% CI, 25.7%-57.3%) in Ohio to a high of 72.9% (95% CI, 67.6%-78.2%) in California (Figure 1).

### Profile of Enrolled Beneficiaries and Unenrolled Individuals Eligible for MSPs

**Figure 2.**
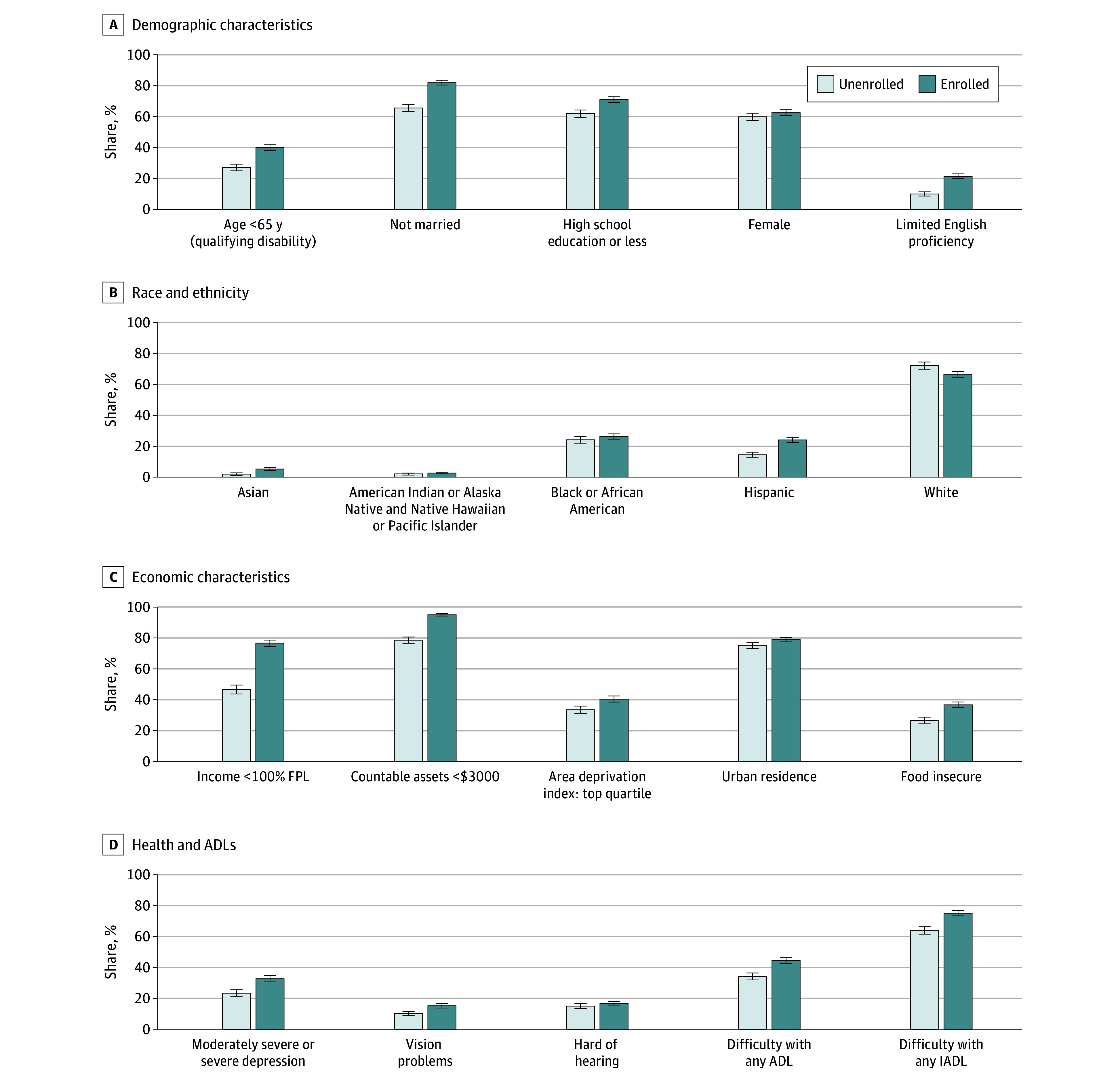
Characteristics of the Enrolled and Unenrolled Populations Among Eligible Beneficiaries Race and ethnicity are self-reported, and survey respondents could choose more than 1 race. We do not report American Indian or Alaska Native and Native Hawaiian or Pacific Islander races separately due to the small sizes and restrictions by the Centers for Medicare & Medicaid Services on minimum required cell sizes for reporting. ADL indicates activity of daily living; FPL, federal poverty level; and IADL, instrumental activity of daily living. Error bars indicate 95% CIs.

Eligible beneficiaries who were enrolled in the MSPs were systematically different in terms of their demographic characteristics, economic well-being, and health compared with those who were eligible but not enrolled (Figure 2). Enrollees were more likely to qualify for Medicare via disability (by 12.8 pp [95% CI, 9.1-16.6 pp]), be unmarried (16.3 pp [95% CI, 12.6-20.0 pp]), have a high school education or less (9.1 pp [95% CI, 4.5-13.7 pp]), and report difficulty speaking English (11.4 pp [95% CI, 8.8-13.9 pp]). Enrollees were also less likely to report being White (−5.6 pp [95% CI, −9.4 to −1.8 pp]) and more likely to report being Asian (3.3 pp [95% CI, 1.8-4.8 pp]) and Hispanic (9.6 pp [95% CI, 6.8-12.5 pp]) than eligible nonenrollees.

The most pronounced differences were economic. Compared with unenrolled eligible individuals, enrollees were 30.0 pp (95% CI, 25.4-34.6 pp) more likely to report incomes under 100% of the FPL and 16.4 pp (95% CI, 13.2-19.6 pp) more likely to report countable assets below $3000 (Figure 2). Health-related differences were also notable. Enrollees were more likely to report moderate or severe depression (9.3 pp [95% CI, 6.0-12.7 pp]) and difficulty in at least 1 activity of daily living (10.4 pp [95% CI, 7.2-13.6 pp]) and in at least 1 instrumental activity of daily living (11.1 pp [95% CI, 7.6-14.6 pp]). All differences are described in eAppendix 6 in Supplement 1.

### Asset Ownership

**Figure 3.**
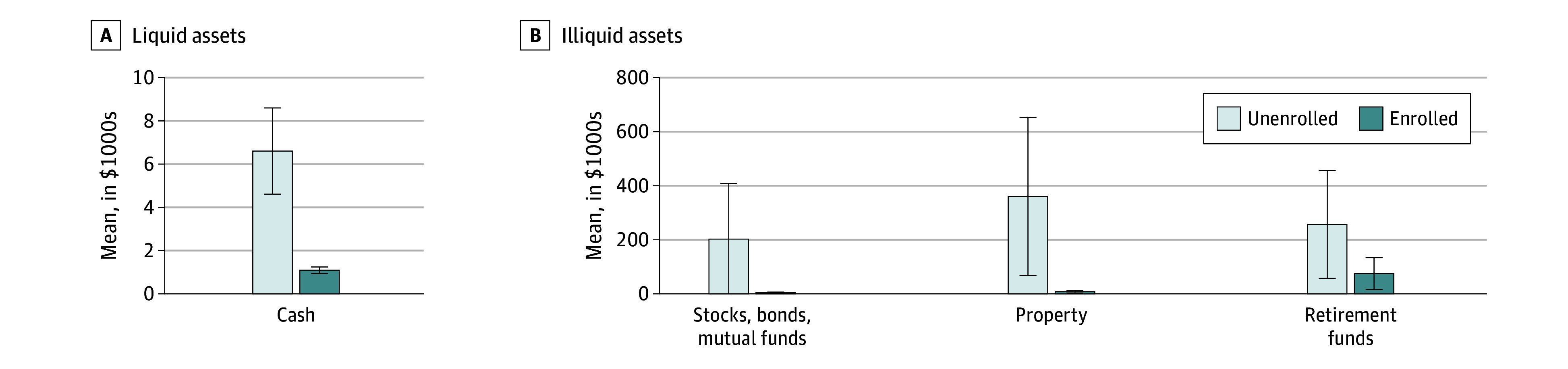
Comparison of Assets Among the Enrolled and Unenrolled Eligible Population, by Asset Type Retirement accounts include those that are in payout status. Property excludes primary residence value, which does not count against Medicare Savings Programs eligibility. Error bars indicate 95% CIs.

Enrolled beneficiaries reported significantly fewer assets than their unenrolled but eligible counterparts. On average, MSP enrollees reported approximately −$5800 (95% CI, −$7900 to −$3700) in liquid cash compared with the unenrolled eligible beneficiaries, –$352 700 (95% CI, –$646 100 to –$59 300) in property value (excluding the primary residence), and –$181 800 (95% CI, –$390 300 to –$26 600) in retirement accounts (Figure 3). Results from models including state fixed effects were similar (eAppendix 6 in Supplement 1).

## Discussion

The MSPs were enacted to defray the financial burden associated with Medicare cost sharing for beneficiaries with low income. This cross-sectional study of a nationally representative sample of community-dwelling Medicare beneficiaries found that 2 of 5 eligible beneficiaries remained unenrolled in the MSPs from 2018 to 2020. This take-up rate of 56.7% is only slightly higher than previous estimates of MSP take-up from 2009 to 2010, nearly a decade earlier and despite several policy efforts since then aimed at increasing MSP enrollment.

One potential explanation for these stagnant MSP take-up rates is the growing popularity of MA plans, which often offer lower premiums and cost sharing and may obviate the need for the MSPs.^24^ Many Medicare beneficiaries with low income report choosing MA specifically because of its potential to reduce their cost-sharing burden, and insurance brokers report recommending MA to enrollees with low income for the same reason.^12,25^ Our finding that MA enrollees are more likely than those in TM to be eligible for the MSPs is consistent with these patterns. However, we found higher MSP enrollment among eligible MA enrollees than among eligible TM enrollees, suggesting MA may not fully substitute for MSP support. Higher enrollment in the MSPs among MA enrollees could also reflect MA plan incentives to encourage MSP enrollment, because MA plans receive higher risk-adjusted payments for beneficiaries enrolled in the MSPs.^26^ At the same time, Medicaid’s “lesser-of” payment rules may reduce cost-sharing reimbursements to provider networks seen by MSP enrollees, making provider networks less willing to accept MSP beneficiaries, which in turn can raise the costs of network formation.^27^ As such, MA plan incentives regarding MSP enrollment remain ambiguous and merit further research.

We found that MSP enrollees were, on average, in poorer health than eligible nonenrollees, including higher rates of disability and self-reported poor health. These associations do not preclude the possibility that certain health conditions may inhibit enrollment. For example, prior research has found that, after accounting for comorbidities, Medicare beneficiaries with poorer cognitive function are less likely to enroll in the MSPs, likely reflecting the administrative complexity of the application and renewal process.^28^

More broadly, our findings show that MSP enrollees tend to be more vulnerable not only in terms of health, but also across socioeconomic dimensions. On one hand, this finding suggests that the MSPs are effectively reaching those most in need. On the other hand, it raises concerns about why eligible individuals who are somewhat better off—but still have low income—are much less likely to enroll in the MSPs. Two structural features of the program may be associated with these patterns and point to policy opportunities.

First, most states have automatic enrollment for recipients of the Supplemental Security Income (SSI), meaning that individuals with particularly limited financial resources—who might otherwise face barriers to participate—are automatically enrolled in the MSPs. Automatic enrollment may increase going forward, as a 2023 CMS rule included a requirement that all states automate MSP enrollment for SSI recipients and screen LIS recipients for likely MSP eligibility.^29^ In contrast, other MSP-eligible beneficiaries who are not enrolled in SSI or the LIS program must actively apply.^30^ In this context, requiring individuals to play an active role in signing up may be taxing and create barriers to enrollment, as individuals with low income face a “bandwidth tax” due to limited monetary and time resources.^31^ Reducing the administrative complexity of enrollment for all eligible individuals by simplifying applications, automating enrollment, and aligning eligibility systems across public programs may further improve MSP take-up, and the effectiveness of these policies will be important to evaluate.

Second, asset testing likely plays a role in deterring participation among eligible individuals with modest financial resources. We found that unenrolled beneficiaries reported higher asset levels than their enrolled counterparts, especially home equity and retirement savings. These assets are harder to document, and because MSP asset tests require detailed documentation of financial resources, beneficiaries with accumulated savings may face more burdensome application processes.^32^ To date, 14 states and the District of Columbia have either eliminated their asset test or enacted policies to do so.^7,33,34,35,36^ Evaluating the association of asset test removal with take-up, especially among individuals with low income who have accumulated some savings, remains a critical area for future research.

Finally, our results do not preclude that some eligible nonenrollees may purposefully forgo the financial support offered by the MSPs. The MSPs are administered by state Medicaid programs, and a study focused on Connecticut found that enrollment in the MSPs limited the beneficiaries’ access to clinicians, potentially because clinicians face administrative difficulties and are reimbursed at lower rates when they engage with Medicaid as a payer for their MSP-enrolled patients.^37^ Given that many states limit clinician reimbursements for MSP patients and impose administrative burdens, policies that remove these disincentives may encourage participation in the MSPs.^38^

### Limitations

Our study had some limitations. First, our data source is representative of community-dwelling Medicare beneficiaries and excludes those residing in nursing homes and other institutional settings. Many long-term care residents spend down their assets to qualify for Medicaid, which in turn may make them eligible for and enrolled in the MSPs, and our results do not generalize to this population. Second, our income and asset information may be subject to measurement error due to recall-based error. Third, although the MCBS is nationally representative, it is not designed to produce precise estimates for smaller subgroups. As a result, our state-level estimates of MSP take-up include only 26 states. Although these states account for most Medicare beneficiaries, the findings may not generalize to smaller states that may be more rural and less bureaucratically robust—factors that could be associated with program implementation and take-up. In addition, the state-level Medicare program type (MA vs TM) and MSP subprogram take-up rates may be limited by sampling variability, small sample sizes, and lack of subgroup representativeness.

## Conclusions

In this cross-sectional study of Medicare beneficiaries, we found that 2 of 5 eligible beneficiaries remained unenrolled in the MSPs and that there was wide variation in take-up across states, ranging from 41.5% to 72.9%. Beneficiaries with the most economic insecurity and with worse health were more likely to be enrolled, suggesting the MSPs are effectively targeted for beneficiaries most in need of support. Policies that reduce administrative barriers to enrollment, improve access to care for MSP enrollees, and promote participation among eligible but unenrolled individuals are likely to increase MSP take-up.

## Notes

Supplement 1. **eAppendix 1.** Federal and State Eligibility Limits During the Study Period **eTable 1.** Federal Limits for the Continental US **eTable 2.** Federal Income Limits for Alaska and Hawaii **eTable 3.** State-Specific Rules in 2018 **eTable 4.** State-Specific Rules in 2019 **eTable 5.** State-Specific Rules in 2020 **eAppendix 2.** Sample Construction and Representativeness **eFigure 1.** Inclusion Criteria **eTable 6.** National Representativeness of the Primary Sample **eAppendix 3.** Calculation of Countable Income and Assets, Validation, and Alternative Eligibility Measures **eTable 7.** Components of the Manually Aggregated Income Measure **eTable 8.** Components of Assets **eFigure 2.** Comparing the Annual Income Variable and the Manually Aggregated Annual Income Variable **eFigure 3.** Comparing the Annual Income Variable and the Manually Aggregated Annual Income, for Households With Annual Income Under $30,000 **eFigure 4.** Sample Asset Value Means and Medians, by Definition **eFigure 5.** Prob(Coded Eligible|Enrolled), by MSP Subprogram and Income and Asset Definition **eAppendix 4.** Covariate Construction and Summary Statistics **eTable 9.** Coding of Basic and Instrumental Activities of Daily Living (ADLs and IADLs) **eTable 10.** Missingness of Covariates Among Eligible Sample, by Enrollment Status **eTable 11.** Eligible Sample Characteristics **eAppendix 5.** Robustness Checks **eFigure 6.** Robustness of Take-Up to Income and Asset Definition **eAppendix 6.** Characteristics of the Enrolled and Unenrolled Eligibles **eTable 12.** Difference in Characteristics of the Enrolled and Unenrolled Eligibles **eTable 13.** Difference in Assets Among the Enrolled and Unenrolled Eligibles **eFigure 7.** Characteristics of the Enrolled and Unenrolled Eligibles, With State Fixed Effects **eFigure 8.** Comparison of Assets Among the Enrolled and Unenrolled Eligibles, With State Fixed Effects
eReferences.

Supplement 2.
Data Sharing Statement

## References

[1] 2024 Medicare Parts A & B premiums and deductibles. Centers for Medicare & Medicaid Services. October 12, 2023. Accessed July 17, 2024. https://www.cms.gov/newsroom/fact-sheets/2024-medicare-parts-b-premiums-and-deductibles?apcid=0063b4a3acf9c7406348c100

[2] Jacobson G, Leonard F. How affordable is health care for Medicare beneficiaries? Commonwealth Fund. November 22, 2024. Accessed August 13, 2025. https://www.commonwealthfund.org/publications/2024/nov/how-affordable-is-health-care-medicare-beneficiaries

[3] Leonard F, Jacobson G, Collins SR, Shah A, Haynes LA. Medicare’s affordability problem: a look at the cost burdens faced by older enrollees. Commonwealth Fund. September 19, 2023. Accessed August 13, 2025. https://www.commonwealthfund.org/publications/issue-briefs/2023/sep/medicare-affordability-problem-cost-burdens-biennial

[4] Rosenbach ML, Lamphere J. Bridging the gaps between Medicare and Medicaid: the case of QMBs and SLMBs. AARP Public Policy Institute. January 1999. Accessed November 28, 2023. https://assets.aarp.org/rgcenter/health/9902_qmbs.pdf

[5] Haber SG, Adamache W, Walsh EG, Hoover S, Bir A. Evaluation of Qualified Medicare Beneficiary (QMB) and Specified Low-Income Medicare Beneficiary (SLMB) programs. RTI International and New England Research Institutes. October 1, 2003. Accessed November 27, 2023. https://www.cms.gov/Research-Statistics-Data-and-Systems/Statistics-Trends-and-Reports/Reports/downloads/haberVol2.pdf

[6] Chapter 4: Medicaid coverage of premiums and cost sharing for low-income Medicare beneficiaries. MACPAC. 2013. Accessed July 17, 2024. https://www.macpac.gov/publication/ch-4-medicaid-coverage-of-premiums-and-cost-sharing-for-low-income-medicare-beneficiaries/

[7] Medicare Savings Programs (MSPs): eligibility and coverage (2020). NCOA. February 2020. Accessed September 13, 2024. https://lists.ncoa.org/bec/cache/13309073/2.pdf

[8] Roberts ET, Glynn A, Cornelio N, . Medicaid coverage “cliff” increases expenses and decreases care for near-poor Medicare beneficiaries. Health Aff (Millwood). 2021;40(4):552-561. doi:10.1377/hlthaff.2020.02272 33819086 PMC8068502

[9] Medicare Savings Programs. Centers for Medicare & Medicaid Services. Accessed July 3, 2025. https://www.medicare.gov/basics/costs/help/medicare-savings-programs

[10] Guidance to states on the low-income subsidy. Centers for Medicare & Medicaid Services. February 2009. Accessed July 3, 2025. https://www.cms.gov/medicare/eligibility-and-enrollment/lowincsubmedicareprescov/downloads/statelisguidance021009.pdf

[11] Ochieng N, Biniek JF. Beneficiary experience, affordability, utilization, and quality in Medicare Advantage and traditional Medicare: a review of the literature. KFF. September 16, 2022. Accessed March 2, 2025. https://www.kff.org/report-section/beneficiary-experience-affordability-utilization-and-quality-in-medicare-advantage-and-traditional-medicare-a-review-of-the-literature-report/

[12] Leonard F, Jacobson G, Haynes LA, Collins SR. Traditional Medicare or Medicare Advantage: how older Americans choose and why. Commonwealth Fund. October 17, 2022. Accessed August 13, 2025. https://www.commonwealthfund.org/publications/issue-briefs/2022/oct/traditional-medicare-or-advantage-how-older-americans-choose

[13] Moon M, Kuntz C, Pounder L. Protecting low income Medicare beneficiaries. Urban Institute. December 1996. Accessed November 28, 2023. https://www.commonwealthfund.org/sites/default/files/documents/___media_files_publications_fund_report_1996_dec_protecting_low_income_medicare_beneficiaries_protect_pdf.pdf

[14] Caswell KJ, Waidmann TA. Medicare Savings Program enrollees and eligible non-enrollees. MACPAC. June 2017. Accessed September 19, 2023. https://www.macpac.gov/wp-content/uploads/2017/08/MSP-Enrollees-and-Eligible-Non-Enrollees.pdf

[15] Rupp K, Sears J. Eligibility for the Medicare buy-in programs, based on a survey of income and program participation simulation. Soc Secur Bull. 2000;63(3):13-25. 11439702

[16] Low-income Medicare beneficiaries: further outreach and administrative simplification could increase enrollment. US General Accounting Office. April 1999. Accessed November 28, 2023. https://www.gao.gov/assets/hehs-99-61.pdf

[17] Keohane LM, Rahman M, Mor V. Reforming access: trends in Medicaid enrollment for new Medicare beneficiaries, 2008-2011. Health Serv Res. 2016;51(2):550-569. doi:10.1111/1475-6773.12349 26251174 PMC4799898

[18] Medicare Savings Programs: implementation of requirements aimed at increasing enrollment. US General Accounting Office. September 2012. Accessed September 19, 2023. https://www.gao.gov/assets/gao-12-871.pdf

[19] Patel SR, Ruggiero DA, Roberts ET. Increasing Medicare Savings Program enrollment—improving affordability of care. JAMA. 2025;333(3):199-200. doi:10.1001/jama.2024.21078 39476128

[20] Medicare Savings Programs (MSPs): eligibility and coverage (2018). NCOA. March 2018. Accessed October 22, 2024. https://www.benavest.com/wp-content/uploads/2019/01/Summary-of-Medicare-Savings-Program-Eligibility-and-Coverage-by-NCOA.pdf

[21] Medicare Savings Programs (MSPs): eligibility and coverage (2019). NCOA. February 2019. Accessed October 22, 2024. https://lists.ncoa.org/mippa/cache/10522477/2.pdf

[22] 2018 MCBS Survey file. Centers for Medicare & Medicaid Services. 2018. Accessed June 25, 2024. https://www.cms.gov/research-statistics-data-and-systemsresearchmcbscodebooks/2018-mcbs-survey-file

[23] Sears J. Comparing beneficiaries of the Medicare savings programs with eligible nonparticipants. Soc Secur Bull. 2001;64(3):76-80. 12655742

[24] Freed M, Biniek JF, Damico A, Neuman T. Medicare Advantage in 2024: enrollment update and key trends. KFF. August 8, 2024. Accessed August 19, 2024. https://www.kff.org/medicare/issue-brief/medicare-advantage-in-2024-enrollment-update-and-key-trends/

[25] Leonard F, Jacobson G, Perry M, Dryden S, Kolb NM. The challenges of choosing Medicare coverage: views from insurance brokers and agents. Commonwealth Fund. February 28, 2023. Accessed August 13, 2025. https://www.commonwealthfund.org/publications/2023/feb/challenges-choosing-medicare-coverage-views-insurance-brokers-agents

[26] Medicare managed care manual: chapter 7—risk adjustment. Centers for Medicare & Medicaid Services. September 19, 2014. Accessed July 3, 2025. https://www.cms.gov/Regulations-and-Guidance/Guidance/Manuals/downloads/mc86c07.pdf

[27] Hayford T, Niu X, Decker S. “Lesser-of” payment policies and the use of physicians’ services among dual-eligible beneficiaries in the United States. Appl Econ. 2025;57(37):5714-5731. doi:10.1080/00036846.2024.2365458

[28] Roberts ET, McGarry BE, Glynn A. Cognition and take-up of the Medicare Savings Programs. JAMA Intern Med. 2020;180(11):1529-1531. doi:10.1001/jamainternmed.2020.2783 33044503 PMC7551226

[29] Streamlining Medicaid; Medicare Savings Program eligibility determination and enrollment. *Federal Register*. September 21, 2023. Accessed August 10, 2024. https://www.federalregister.gov/documents/2023/09/21/2023-20382/streamlining-medicaid-medicare-savings-program-eligibility-determination-and-enrollment

[30] Medicaid information. Social Security Administration. Accessed April 9, 2025. https://www.ssa.gov/disabilityresearch/wi/medicaid.htm

[31] Bearson DF, Sunstein CR. Take up. Behavioural Public Policy. Published online October 4, 2023. doi:10.1017/bpp.2023.21

[32] Glaun K. Medicaid programs to assist low-income Medicare beneficiaries: working paper on Medicare Savings Programs in Connecticut. Henry J. Kaiser Family Foundation. December 2002. Accessed November 28, 2023. https://www.kff.org/wp-content/uploads/2013/01/medicaid-programs-to-assist-low-income-medicare-beneficiaries-working-paper-on-medicare-savings-programs-in-connecticut-background-paper.pdf

[33] Asset limit changes for non-MAGI Medi-Cal. Department of Health Care Services, State of California. 2023. Accessed September 13, 2024. https://www.dhcs.ca.gov/services/medi-cal/eligibility/Pages/Asset-Limit-Changes-for-Non-MAGI-Medi-Cal.aspx

[34] Rossi H. MassHealth: eligibility operations memo 24-03. Commonwealth of Massachusetts, Executive Office of Health and Human Services, Office of Medicaid. March 2024. Accessed September 13, 2024. https://www.mass.gov/doc/eom-24-03-medicare-savings-programs-formerly-known-as-masshealth-senior-buy-in-and-masshealth-buy-in-programs-0/download

[35] Westenhaver M. Medicaid state plan amendment (SPA) 22-0034: Medicare Savings Program. Health Care Authority, State of Washington. October 11, 2022. Accessed September 13, 2024. https://www.hca.wa.gov/assets/22-0034-MedicareSavingsProgramPublicNotice-WSR-22-21-048.pdf

[36] MaineCare rule #304—CHIP coverage group change and Medicare Savings Program change. Department of Health and Human Services, State of Maine. May 29, 2024. Accessed November 27, 2024. https://www.maine.gov/dhhs/about/rulemaking/mainecare-rule-304-chip-coverage-group-change-and-medicare-savings-program-change-2024-05-29

[37] Li G. Becoming dual: measuring the impact of gaining Medicaid coverage for Medicare beneficiaries. June 2023. Accessed September 18, 2023. https://drive.google.com/file/d/1Ih54083fNxiAd1bTBAyTZ6ZvmMfGKU2H/view

[38] Roberts ET, Nimgaonkar A, Aarons J, . New evidence of state variation in Medicaid payment policies for dual Medicare-Medicaid enrollees. Health Serv Res. 2020;55(5):701-709. doi:10.1111/1475-6773.13545 33460128 PMC7518808

