# Supplementary material for: Medicare Savings Program Take-Up Estimates and Profile of Enrolled and Unenrolled Individuals
Source: JAMA Netw Open. 2025 Oct 3;8(10):e2535408. doi: 10.1001/jamanetworkopen.2025.35408 (PMC12495494; doi:10.1001/jamanetworkopen.2025.35408)
Supplement: Supplement 1. — eAppendix 1. Federal and State Eligibility Limits During the Study Period eTable 1. Federal Limits for the Continental US eTable 2. Federal Income Limits for Alaska and Hawaii eTable 3. State-Specific Rules in 2018 eTable 4. State-Specific Rules in 2019 eTable 5. State-Specific Rules in 2020 eAppendix 2. Sample Construction and Representativeness eFigure 1. Inclusion Criteria eTable 6. National Representativeness of the Primary Sample eAppendix 3. Calculation of Countable Income and Assets, Validation, and Alternative Eligibility Measures eTable 7. Components of the Manually Aggregated Income Measure eTable 8. Components of Assets eFigure 2. Comparing the Annual Income Variable and the Manually Aggregated Annual Income Variable eFigure 3. Comparing the Annual Income Variable and the Manually Aggregated Annual Income, for Households With Annual Income Under $30,000 eFigure 4. Sample Asset Value Means and Medians, by Definition eFigure 5. Prob(Coded Eligible|Enrolled), by MSP Subprogram and Income and Asset Definition eAppendix 4. Covariate Construction and Summary Statistics eTable 9. Coding of Basic and Instrumental Activities of Daily Living (ADLs and IADLs) eTable 10. Missingness of Covariates Among Eligible Sample, by Enrollment Status eTable 11. Eligible Sample Characteristics eAppendix 5. Robustness Checks eFigure 6. Robustness of Take-Up to Income and Asset Definition eAppendix 6. Characteristics of the Enrolled and Unenrolled Eligibles eTable 12. Difference in Characteristics of the Enrolled and Unenrolled Eligibles eTable 13. Difference in Assets Among the Enrolled and Unenrolled Eligibles eFigure 7. Characteristics of the Enrolled and Unenrolled Eligibles, With State Fixed Effects eFigure 8. Comparison of Assets Among the Enrolled and Unenrolled Eligibles, With State Fixed Effects eReferences. [file jamanetwopen-e2535408-s001.pdf]

Supplementary Online Content

Kotb S, Su A, Sinaiko AD. Medicare Savings Program take-up estimates and profile of enrolled and unenrolled individuals. *JAMA Netw Open.* 2025;8(10):e2535408. doi:10.1001/jamanetworkopen.2025.35408

eAppendix 1. Federal and State Eligibility Limits During the Study Period
eTable 1. Federal Limits for the Continental US
eTable 2. Federal Income Limits for Alaska and Hawaii
eTable 3. State-Specific Rules in 2018
eTable 4. State-Specific Rules in 2019
eTable 5. State-Specific Rules in 2020
eAppendix 2. Sample Construction and Representativeness
eFigure 1. Inclusion Criteria
eTable 6. National Representativeness of the Primary Sample
eAppendix 3. Calculation of Countable Income and Assets, Validation, and Alternative Eligibility Measures
eTable 7. Components of the Manually Aggregated Income Measure
eTable 8. Components of Assets
eFigure 2. Comparing the Annual Income Variable and the Manually Aggregated Annual Income Variable
eFigure 3. Comparing the Annual Income Variable and the Manually Aggregated Annual Income, for Households With Annual Income Under $30,000
eFigure 4. Sample Asset Value Means and Medians, by Definition
eFigure 5. Prob(Coded Eligible|Enrolled), by MSP Subprogram and Income and Asset Definition
eAppendix 4. Covariate Construction and Summary Statistics
eTable 9. Coding of Basic and Instrumental Activities of Daily Living (ADLs and IADLs)
eTable 10. Missingness of Covariates Among Eligible Sample, by Enrollment Status
eTable 11. Eligible Sample Characteristics
eAppendix 5. Robustness Checks
eFigure 6. Robustness of Take-Up to Income and Asset Definition
eAppendix 6. Characteristics of the Enrolled and Unenrolled Eligibles
eTable 12. Difference in Characteristics of the Enrolled and Unenrolled Eligibles
eTable 13. Difference in Assets Among the Enrolled and Unenrolled Eligibles
eFigure 7. Characteristics of the Enrolled and Unenrolled Eligibles, With State Fixed Effects
eFigure 8. Comparison of Assets Among the Enrolled and Unenrolled Eligibles, With State Fixed Effects
eReferences.

© 2025 Kotb et al. *JAMA Network Open.*

This supplementary material has been provided by the authors to give readers additional information about their work.

© 2025 Kotb et al. *JAMA Network Open.*

eAppendix 1. Federal and State Eligibility Limits During the Study Period

This section summarizes state eligibility rules for the Medicare Savings Programs from 2018 to 2020. We use this information to measure whether each beneficiary in the sample is eligible for the MSPs.

eTable 1. Federal Limits for the Continental US

Year	Monthly Income Eligibility Before Disregards	Asset Eligibility	States
2018	Federal income standards:100% FPL for QMB, 101-120% FPL for SLMB, 121-135% for QI:<br>• QMB: $1012 single, $1372 married<br>• SLMB: $1214 single, $1646 married<br>• QI: $1366 single, $1852 married	$7560 single, $11340 married	Arkansas, California, Colorado, Florida, Georgia, Idaho, Iowa, Kansas, Kentucky, Louisiana, Maryland, Massachusetts, Michigan, Missouri, Montana, Nebraska, Nevada, New Hampshire, New Jersey, New Mexico, North Carolina, North Dakota, Ohio, Oklahoma, Pennsylvania, Rhode Island, South Carolina, South Dakota, Tennessee, Texas, Utah, Virginia, Washington, West Virginia, Wisconsin, Wyoming
2019	Federal income standards:100% FPL for QMB, 101-120% FPL for SLMB, 121-135% for QI:<br>• QMB: $1041 single, $1410 married<br>• SLMB: $1249 single, $1691 married<br>• QI: $1406 single, $1903 married	$7730 single, $11600 married	Arkansas, California, Colorado, Florida, Georgia, Idaho, Iowa, Kansas, Kentucky, Louisiana, Maryland, Massachusetts, Michigan, Missouri, Montana, Nebraska, Nevada, New Hampshire, New Jersey, New Mexico, North Carolina, North Dakota, Ohio, Oklahoma, Pennsylvania, Rhode Island, South Carolina, South Dakota, Tennessee, Texas, Utah, Virginia, Washington, West Virginia, Wisconsin, Wyoming
2020	Federal income standards:100% FPL for QMB, 101-120% FPL for SLMB, 121-135% for QI:<br>• QMB: $1063 single, $1437 married<br>• SLMB: $1276 single, $1724 married<br>• QI: $1436 single, $1940 married	$7860 single, $11800 married	Arkansas, California, Colorado, Florida, Georgia, Idaho, Iowa, Kansas, Kentucky, Maryland, Michigan, Missouri, Montana, Nebraska, Nevada, New Hampshire, New Jersey, New Mexico, North Carolina, North Dakota, Ohio, Oklahoma, Pennsylvania, Rhode Island, South Carolina, South Dakota, Tennessee, Texas, Utah, Virginia, Washington, West Virginia, Wisconsin, Wyoming

Sources: NCOA.[1–3] *Notes:* Between 2019 and 2020, three states – Louisiana, Massachusetts, and Maine – increased their income and/or asset limit for program eligibility.[4–6] Effective March 2020, Illinois temporarily suspended their asset test during the Public Health Emergency.[7]

© 2025 Kotb et al. *JAMA Network Open.*

eTable 2. Federal Income Limits for Alaska and Hawaii

State	Year	Monthly Income Eligibility Before Disregards
Alaska	2018	Federal income standards:100% FPL for QMB, 101-120% FPL for SLMB, 121-135% for QI:<br>• QMB: $1265 single, $1715 married<br>• SLMB: $1518 single, $2058 married<br>• QI: $1708 single, $2316 married
	2019	Federal income standards:100% FPL for QMB, 101-120% FPL for SLMB, 121-135% for QI:<br>• QMB: $1300 single, $1761 married<br>• SLMB: $1560 single, $2113 married<br>• QI: $1755 single, $2378 married
	2020	Federal income standards:100% FPL for QMB, 101-120% FPL for SLMB, 121-135% for QI:<br>• QMB: $1329 single, $1796 married<br>• SLMB: $1595 single, $2155 married<br>• QI: $1794 single, $2425 married
Hawaii	2018	Federal income standards:100% FPL for QMB, 101-120% FPL for SLMB, 121-135% for QI:<br>• QMB: $1164 single, $1578 married<br>• SLMB: $1396 single, $1893 married<br>• QI: $1571 single, $2130 married
	2019	Federal income standards:100% FPL for QMB, 101-120% FPL for SLMB, 121-135% for QI:<br>• QMB: $1199 single, $1622 married<br>• SLMB: $1438 single, $1946 married<br>• QI: $1618 single, $2190 married
	2020	Federal income standards:100% FPL for QMB, 101-120% FPL for SLMB, 121-135% for QI:<br>• QMB: $1223 single, $1653 married<br>• SLMB: $1468 single, $1983 married<br>• QI: $1652 single, $2231 married

Notes: Asset eligibility in Alaska and Hawaii are the same as those in Continental U.S.

© 2025 Kotb et al. *JAMA Network Open.*

eTable 3. State-Specific Rules in 2018

States	Monthly Income Eligibility (Including Any Disregards)	Asset Eligibility	Notes
Alabama	Federal rule	No limit	
Arizona	Federal rule	No limit	
Connecticut	• QMB: $2120.55 single, $2854.83 married<br>• SLMB: $2321.55 single, $3125.43 married<br>• QI: $2472.30 single, $3328.38 married	No limit	CT uses the 2017 eligibility rules until 7/1/2018 (per HB 7601). Income limits are 211/231/246% FPL with no disregard
Delaware	Federal rule	No limit	
District of Columbia	• QMB: $3035 single, $4115 married	No limit	Income limit is 300% FPL with no disregard. DC only has QMB program
Illinois	• QMB: $1037 single, $1397 married<br>• SLMB: $1239 single, $1671 married<br>• QI: $1391 single, $1877 married	Federal rule	Income disregard is $25 per household
Indiana	• QMB: $1518 single, $2058 married<br>• SLMB: $1720 single; $2332 married<br>• QI: $1872 single, $2538 married	Federal rule	Income limits are 150/170/185% FPL
Maine	• QMB: $1492 single, $2021 married<br>• SLMB: $1694 single, $2295 married<br>• QI: $1846 single, $2501 married	$58000 liquid assets single, $87000 liquid assets married	Income disregard is $75 single / $100 couple. Income limits are 140/160/175% FPL
Minnesota	Federal rule	$10000 single, $18000 married	
Mississippi	• QMB: $1062 single, $1422 married<br>• SLMB: $1264 single, $1696 married<br>• QI: $1416 single, $1902 married	No limit	Income disregard is $50 per household
New York	Federal rule	No limit	
Oregon	Federal rule	No limit	
Vermont	Federal rule	No limit	

Notes: The state of Maine defines liquid assets as including “cash or other resources that can be changed into cash on demand like cash value of life insurance.”[8,9]

© 2025 Kotb et al. *JAMA Network Open.*

eTable 4. State-Specific Rules in 2019

States	Monthly Income Eligibility (Including Any Disregards)	Asset Eligibility	Notes
Alabama	Federal rule	No limit	
Arizona	Federal rule	No limit	
Connecticut	• QMB: $2196 single, $2973 married<br>• SLMB: $2404 single, $3255 married<br>• QI: $2560 single, $3467 married	No limit	Income limits are 211/231/246% FPL with no disregard
Delaware	Federal rule	No limit	
District of Columbia	• QMB: $3123 single, $4228 married	No limit	Income limit is 300% FPL with no disregard. DC only has QMB program
Illinois	• QMB: $1066 single, $1435 married<br>• SLMB: $1274 single, $1716 married<br>• QI: $1431 single, $1928 married	Federal rule	Income disregard is $25 per household
Indiana	• QMB: $1562 single, $2115 married<br>• SLMB: $1770 single, $2397 married<br>• QI: $1926 single, $2609 married	Federal rule	Income limits are 150/170/185% FPL
Maine	• QMB: $1532 single, $2074 married<br>• SLMB: $1741 single, $2356 married<br>• QI: $1897 single, $2568 married	$58000 liquid assets single, $87000 liquid assets married	Income disregard is $75 single / $100 couple. Income limits are 140/160/175% FPL
Minnesota	Federal rule	$10000 single, $18000 married	
Mississippi	• QMB: $1091 single, $1460 married<br>• SLMB: $1299 single, $1749 married<br>• QI: $1456 single, $1953 married	No limit	Income disregard is $50 per household
New York	Federal rule	No limit	
Oregon	Federal rule	No limit	
Vermont	Federal rule	No limit	

© 2025 Kotb et al. *JAMA Network Open.*

eTable 5. State-Specific Rules in 2020

States	Monthly Income Eligibility (Including Any Disregards)	Asset Eligibility	Notes
Alabama	Federal rule	No limit	
Arizona	Federal rule	No limit	
Connecticut	• QMB: $2245 single, $3032 married<br>• SLMB: $2458 single, $3319 married<br>• QI: $2617 single, $3535 married	No limit	Income limits are 211/231/246% FPL with no disregard
Delaware	Federal rule	No limit	
District of Columbia	• QMB: $3190 single, $4310 married	No limit	Income limit is 300% FPL with no disregard. DC only has QMB program
Illinois	• QMB: $1088 single, $1462 married<br>• SLMB: $1301 single, $1749 married<br>• QI: $1461 single, $1965 married	Asset test suspended effective March 2020 due to PHE. Federal rule prior to March.	Income disregard is $25 per household<br><br>Effective 3/1/2020: also temporarily suspended asset test due to PHE
Indiana	• QMB: $1595 single, $2155 married<br>• SLMB: $1807 single, $2443 married<br>• QI: $1967 single, $2658 married	Federal rule	Income limits are 150/170/185% FPL
Louisiana	Federal rule	No limit	
Maine	• QMB: $1670 single, $2255 married<br>• SLMB: $1882 single, $2543 married<br>• QI: $2043 single, $2758 married	$58000 liquid assets single, $87000 liquid assets married	Income disregard is $75 single / $100 couple. Income limits increased to 150/170/185% FPL.
Massachusetts	• QMB: $1382 single, $1868 married<br>• SLMB: $1595 single, $2156 married<br>• QI: $1754 single, $2371 married	$15720 single, $23600 married	Income limits are 130/150/165% FPL
Minnesota	Federal rule	$10000 single, $18000 married	
Mississippi	• QMB: $1113 single, $1487 married	No limit	Income disregard is $50 per household

© 2025 Kotb et al. *JAMA Network Open.*

	• SLMB: $1326 single, $1774 married<br>• QI: $1486 single, $1990 married	
New York	Federal rule	No limit
Oregon	Federal rule	No limit
Vermont	Federal rule	No limit

© 2025 Kotb et al. *JAMA Network Open.*

eAppendix 2. Sample Construction and Representativeness

The primary sample includes all MCBS respondents who were asked to complete the income and asset questionnaire. There were 26,240 observations in the primary sample across the three years, representing 170,221,355 beneficiary-years. The eligible sample represents the enrollees within the primary sample who were deemed eligible according to our method. These include 6,608 observations, representing 35,587,360 beneficiary years. The following diagram represents the steps taken to reach these two samples.

eFigure 1. Inclusion Criteria

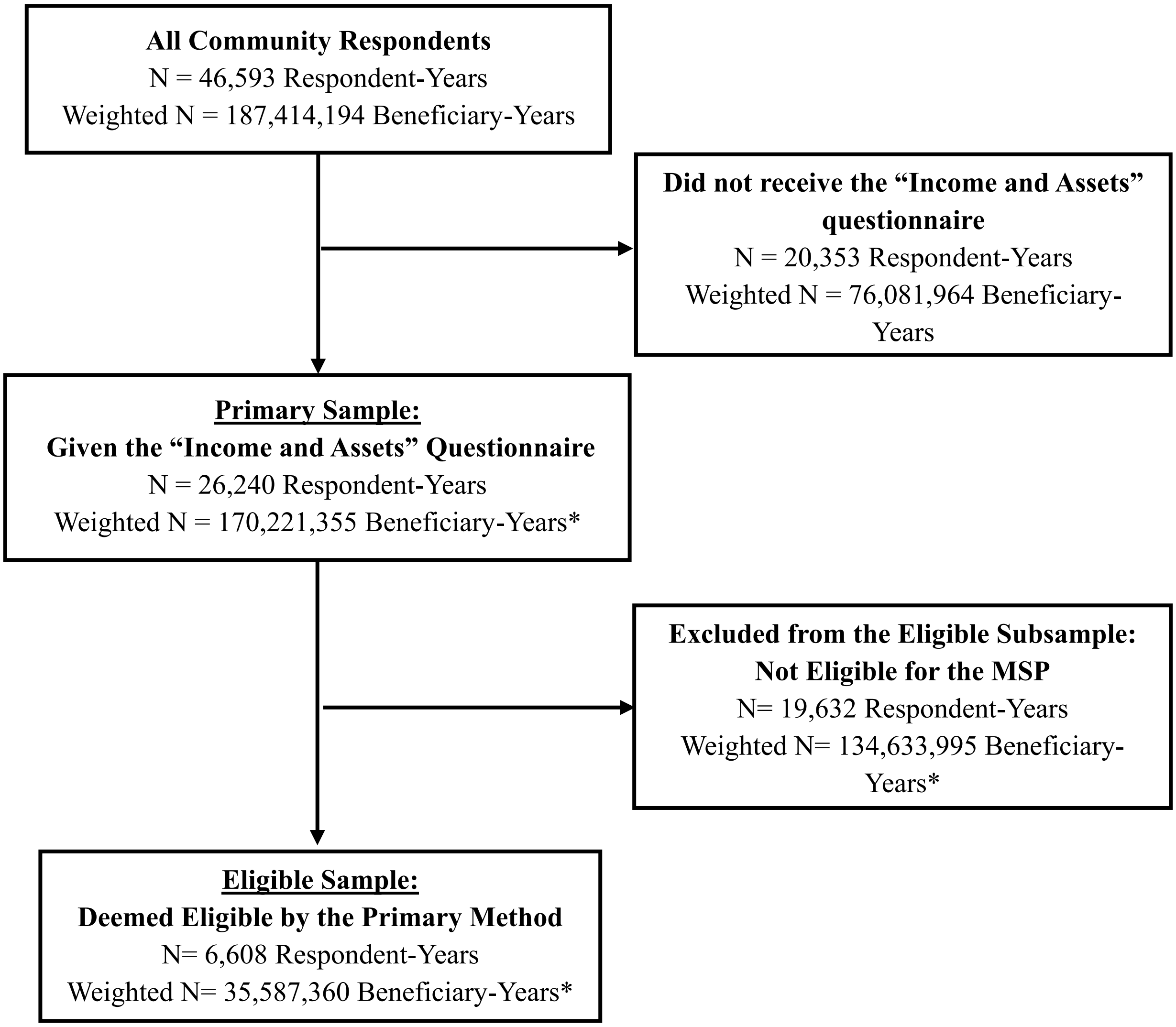

Notes: *Re-weighted using survey weights that are different from those used in the first box to make respondents to the income and asset questionnaire (i.e., the primary sample) nationally representative of all ever-enrolled, community-dwelling Medicare beneficiaries in a given year.

© 2025 Kotb et al. *JAMA Network Open.*

eTable 6. National Representativeness of the Primary Sample

	(1) Primary Sample (Income and Asset Questionnaire Respondents) (%)	(2) All Community Respondents (%)
Demographic Characteristics		
Age Under 65 (Qualified through a disability)	13.96	13.73
Not Married	47.13	48.62
High School Degree or Lower	37.66	40.04
Female	55.05	54.48
Limited English Proficiency	4.69	4.87
Race and Ethnicity		
White	85.51	84.85
Black or African American	11.04	11.25
Asian	2.78	3.05
AIAN and NHPI	1.16	1.32
Hispanic Ethnicity	8.3	8.42
Economic Characteristics		
Income under 100% FPL	14.76	15.81
Assets under $3,000	32.78	59.91
Area Deprivation Index: Top Quartile	19.95	20.29
Urban residence	81.07	80.76
Food insecure	12.06	11.96
	14.76	15.81
Health and Daily Activities		
Moderately Severe or Severe Depression	15.75	16.31
Vision Problems	7.51	8.1
Hard of Hearing	15.8	16.46
Difficulty in Any Activity of Daily Living	25.63	26.78
Difficulty in Any Instrumental Activity of Daily Living	53.53	54.3
Person-Years	170,221,355	187,414,194

Notes: Column (1) uses weights specific to the income and asset questionnaire in the MCBS, which re-weighs the respondents who were asked to complete this part of the survey and make them nationally representative of the ever-enrolled population and adjust for non-response. Column (2) uses the MCBS-provided weights for the ever-enrolled community-dwelling population in Medicare. Race is self-reported and survey respondents could choose more than one race. We do not report AIAN and NHPI separately due to small samples and CMS restrictions on minimum required cell sizes for reporting. NHPI indicates Native Hawaiian or Pacific Islander race; AIAN, American Indian or Alaska Native race; ADL indicates activity of daily living; and IADL, instrumental activity of daily living.

© 2025 Kotb et al. *JAMA Network Open.*

Survey Sample Weights:

For both our primary sample (i.e. Income and Asset questionnaire respondents) and our eligible sample (those deemed eligible as described in eAppendix 3 and the manuscript), we use weights specific to the Income and Asset section of the survey. These weights account for the nonresponse rates among respondents who answered income and asset questions each round as well as the non-random sampling of households for the surveys. These weights are positive for any community-dwelling Medicare beneficiaries who answered the income and assets questionnaire and who were ever enrolled during the calendar year.

© 2025 Kotb et al. *JAMA Network Open.*

eAppendix 3. Calculation of Countable Income and Assets, Validation, and Alternative Eligibility Measures

Methods for Income and Asset Calculation:

In our primary income measure, we start with the MCBS-provided variable ("*income_h*") that captures total household annual income, then determine the monthly countable income by applying the program adjustments (e.g., wage deductions; Part B premium deductions, etc. More detail about these adjustments is provided in the next section). The monthly countable income is the measure of income used to determine MSP eligibility. To assess the sensitivity of our results to our primary income measure, we also construct a secondary income measure that we create by manually aggregating the individual components reported in eTable 7 and applying program adjustments.

eTable 7. Components of the Manually Aggregated Income Measure

Component	Variable name in the MCBS	Notes
Unearned Income		
Monthly SSI	*ssi*	Multiplied by 12
Monthly VA payment	*va*	Multiplied by 12
Monthly Pension	*pension*	Multiplied by 12
Social Security retirement and/or Railroad Retirement payments	*ssrr*	Multiplied by the number of months received in year
Retirement	*ly401k*	
Income from other assets	*landinc*	
Any lumpsum income from inheritance, a trust fund, an insurance settlement, and so on	*lumpamt*	
Earned Income		
Wage	*work*	Multiplied by 12

Note that some sources of countable income are not explicitly inquired about in the survey. These include public assistance, unemployment income, insurance payments, worker compensation, alimony payment, and any commissions.[10] To account for these particular sources of income, we use the lumpsum income variable ("*lumpamt*").

Our preferred income measure is the total household annual income variable ("*income_h*"), partly because of this exclusion of some income sources in our manually aggregated income measure. More importantly, our primary measure is more reliable given that many of the individual components in the manually aggregated income measure were inquired about with a reference period reflecting the month prior to the questionnaire rather than the year in question, meaning that our primary measure will capture relatively less noise.

© 2025 Kotb et al. *JAMA Network Open.*

Methods for Asset Calculation:

We calculate countable assets by adding the value of any owned property (excluding real estate that counts as primary residence), any liquid assets in a bank account, and illiquid stocks, mutual funds, and bonds. We also include retirement accounts that are not in payout status as part of an individual's asset value. The specific variables that we include in our calculation of countable assets are described below:

eTable 8. Components of Assets

Component	Variable name in the MCBS	Notes
Business, farm, real estate (other than primary residence)	*land*	
Savings (checking accounts, saving accounts, money market accounts, certificates of deposits)	*bank*	
Stocks, bonds, and mutual funds	*fund*	
Retirement Accounts	*tot401k*	To determine whether an account is in payout status we use the variable: *ly401k*

Validation for Income and Assets:

To validate our primary MCBS-provided income measure ("*income_h*"), we compare it against a manually aggregated measure of annual income. As eFigure 2 shows, the two income measures have roughly similar distributions. eFigure 3 zooms in on households that have a total annual income under $30,000 that may be more likely to be eligible for the MSPs.

For each of the two income measures, we then proceed to calculate two definitions of the household's *countable* monthly income. In our primary measure of income, we assume that the MCBS-provided income variable includes earned and unearned income; we then subtract annual wages from it, and add the countable wage and annual Medicare Part B premiums. We note that the wage variable is derived from a survey question with a different recall period (month prior to the survey instead of the year in question). As such, it may include some measurement error if people's wages fluctuate, though we note that only 22% reported a non-zero wage in a given year.

For our manually aggregated measure of income, we include half of the monthly wage net of $65, any premium paid by the household towards Part B of Medicare (*h_ptbprm*), and any unearned income. Part B premium was multiplied by two if both the individual and their spouse received retirement benefits. For both these countable income measures, we apply the federal or state-specific disregards as shown in eTables 1 through 5.

© 2025 Kotb et al. *JAMA Network Open.*

eFigure 2. Comparing the Annual Income Variable and the Manually Aggregated Annual Income Variable

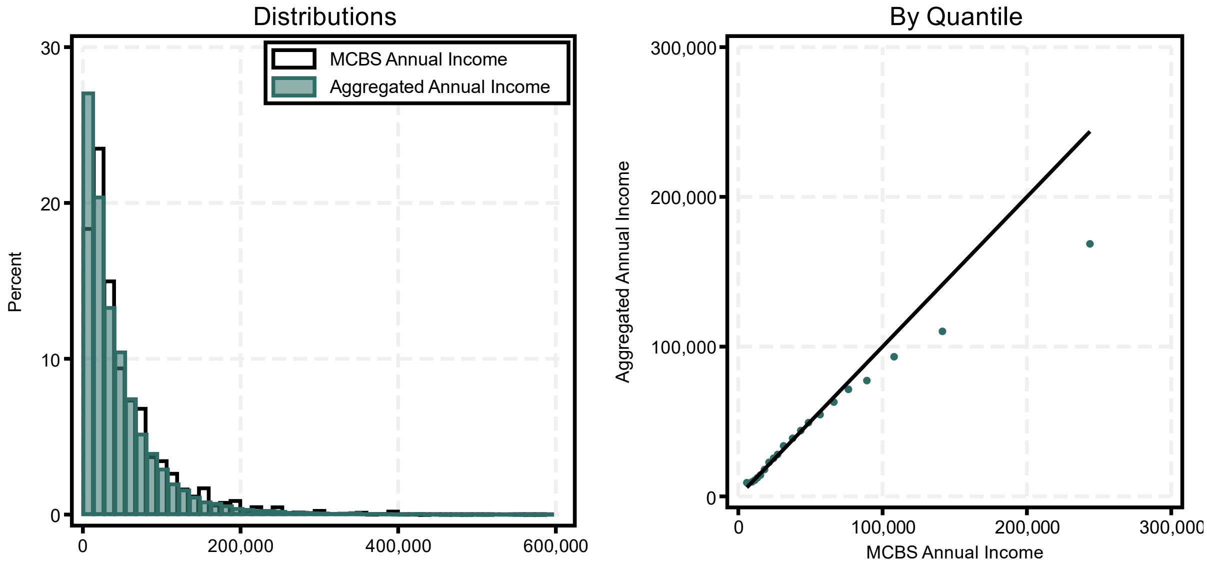

Both graphs censor annual income at $600,000.

© 2025 Kotb et al. *JAMA Network Open.*

eFigure 3. Comparing the Annual Income Variable and the Manually Aggregated Annual Income, for Households With Annual Income Under $30,000

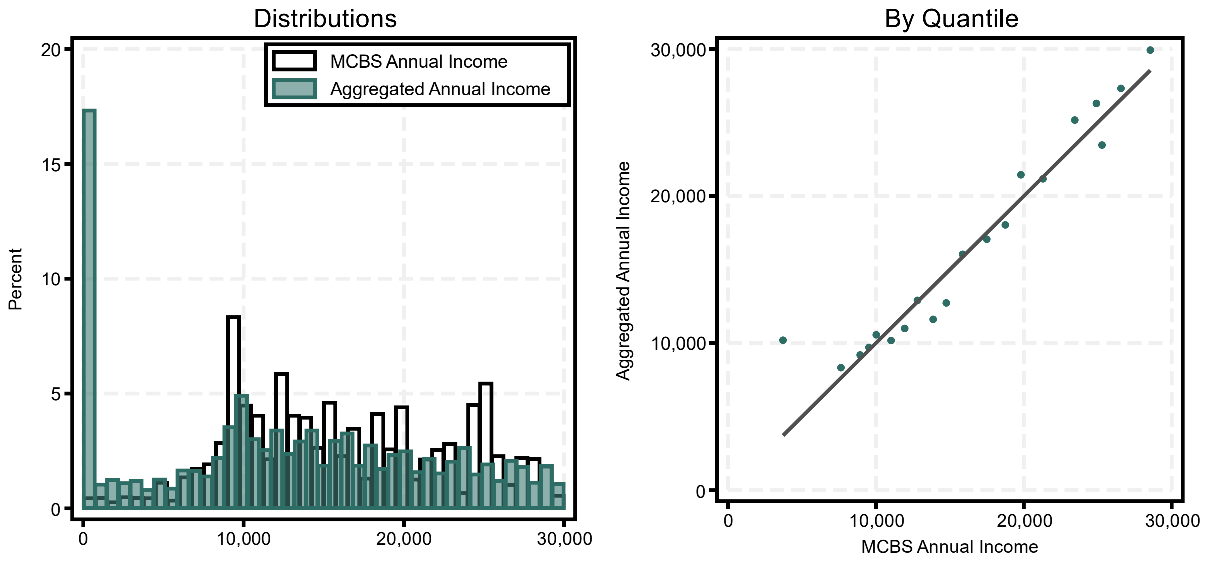

We also consider two alternative definitions of countable assets based on retirement accounts: one in which none of it is included and one in which all of it is included. eFigure 4 presents the means and median values of these two definitions and the primary one we use in the manuscript and in the primary analyses.

Much like the individual component income variables, some asset types are also reported at a given point in the year following the survey year, imposing measurement error. Given that assets are likely to appreciate, our eligibility measure is likely to be underestimated, and our take-up results are therefore likely to be conservative.

© 2025 Kotb et al. *JAMA Network Open.*

eFigure 4. Sample Asset Value Means and Medians, by Definition

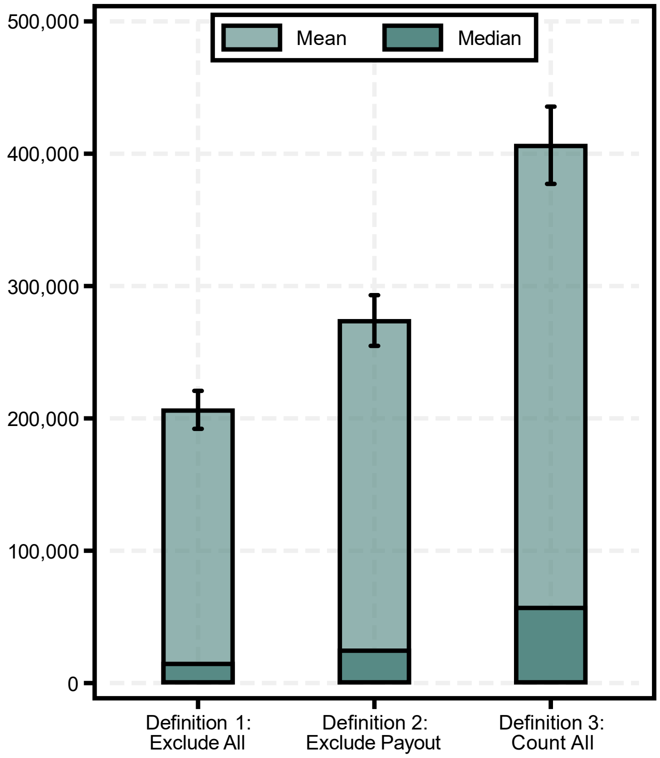

© 2025 Kotb et al. *JAMA Network Open.*

Sensitivity to Income and Asset Construction:

eFigure 5. Prob(Coded Eligible|Enrolled), by MSP Subprogram and Income and Asset Definition

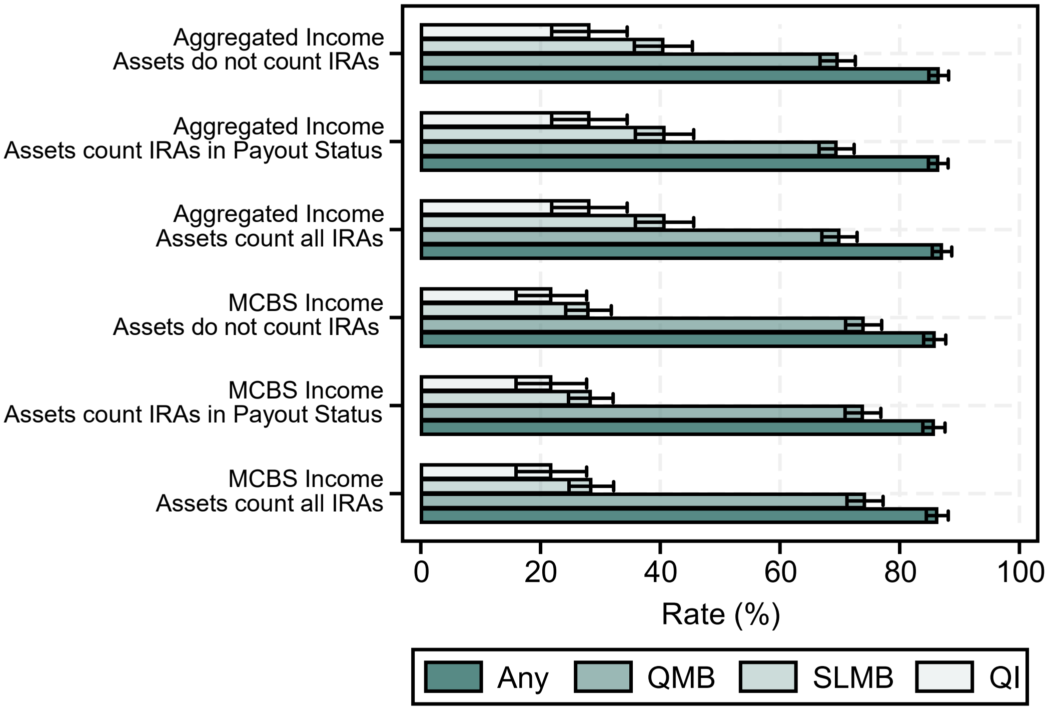

Source: Authors' analyses of the MCBS data linked to enrollment from Medicare data for the years 2018 through 2020.

Notes: The graph presents the share of MSP enrollees who were classified as eligible using our method, by the MSP subprogram. QMB refers to the Qualified Medicare Beneficiary program, SLMB refers to the Specified Low-Income Medicare Beneficiary program, and QI refers to the Qualifying Individual program.

© 2025 Kotb et al. *JAMA Network Open.*

eAppendix 4. Covariate Construction and Summary Statistics

eTable 9. Coding of Basic and Instrumental Activities of Daily Living (ADLs and IADLs)

Survey question	MCBS variable	Coding
Difficulty with activities of daily living		
Any difficulty walking?	*hppdwalk*	ADL = 1 if the MCBS variable has a value of 1 (Yes) or 3 (Doesn't do)
Any difficulty get in/out of bed/chair?	*hppdchar*	
Any difficulty dressing?	*hppddres*	
Any difficulty eating?	*hppdeat*	
Any difficulty bathing/showering?	*hppdbath*	
Any difficulty using the toilet?	*hppdtoil*	
Difficulty with instrumental activities of daily living		
Any difficulty using telephone?	*prbtele*	IADL = 1 if the MCBS variable has a value of 1 (Yes) or 3 (Doesn't do)
Any difficulty shopping?	*prbshop*	
Any difficulty with managing money?	*prbbils*	
Any difficulty doing light/heavy housework?	*prblhwk, prbhhwk*	
Any difficulty preparing meals?	*prbmeal*	
Difficulty stooping/crouching/kneeling	*difstoop*	IADL = 1 if the MCBS variable has a value of 3 (Some difficulty), 4 (A lot of difficulty), or 5 (Not able to do it)
Difficulty lifting/carrying 10 pounds	*diflift*	
Difficulty extending arms above shoulder	*difreach*	
Difficulty writing/handling object	*difwrite*	
Difficulty doing errands	*diserrnd*	IADL = 1 if the MCBS variable = 1 (Yes)

Notes: For all of these survey questions, the activities of daily living (ADLs) or instrumental activities of daily living (IADLs) are coded as missing if the variable has a value of ".", ".d" (don't know), ".r" (refused). We also code the aggregated "any ADL" or "any IADL" variables as missing if any subcomponent is also missing.

© 2025 Kotb et al. *JAMA Network Open.*

Missingness of Covariates For the eligible sample, we tabulated the share of observations that are missing. eTable 10 shows the results below.

eTable 10. Missingness of Covariates Among Eligible Sample, by Enrollment Status

	(1)	(2)	(3)
	Percent Missing, unweighted (%)		
	All Eligible Sample	Enrolled	Unenrolled
Demographic Characteristics	1.0	1.1	0.9
Age Under 65 (Qualified through a disability)	0.0	0.0	0.0
Not Married	0.0	0.0	0.0
High School Degree or Lower	0.8	0.8	0.7
Female	0.0	0.0	0.0
Limited English Proficiency	0.4	0.5	*
Race and Ethnicity	5.9	5.9	5.8
White	5.3	5.1	5.1
Black or African American	5.3	5.1	5.1
Asian	5.3	5.1	5.1
AIAN and NHPI	5.3	5.1	5.1
Hispanic Ethnicity	0.6	0.8	0.8
Economic Characteristics	36.7	37.5	35.5
Income under 100% FPL	30.8	30.8	30.9
Assets under $3,000	0.0	0.0	0.0
Area Deprivation Index – Top Quartile	6.9	7.6	5.7
Urban residence	*	*	*
Food insecure	0.7	0.9	0.5
Health and Daily Activities	21.9	24.4	17.8
Moderately Severe or Severe Depression	20.7	23.4	16.5
Vision Problems	1.3	1.5	1.0
Hard of Hearing	1.3	1.4	1.0
Difficulty in Any Activity of Daily Living	1.8	1.9	1.7
Difficulty in Any Instrumental Activity of Daily Living	3.0	3.1	2.8

Notes: The three columns show the share of observations that were missing a given covariate. Race is self-reported and survey respondents could choose more than one race. Race is self-reported and survey respondents could choose more than one race. We do not report AIAN and NHPI separately due to small samples and CMS restrictions on minimum required cell sizes for reporting. NHPI indicates Native Hawaiian or Pacific Islander race; AIAN, American Indian or Alaska Native race; ADL indicates activity of daily living; and IADL, instrumental activity of daily living. * indicates a censored cell due to the presence of either a low numerator or a low denominator.

For analyses comparing the prevalence of characteristics between enrolled and unenrolled eligible individuals (e.g., Figure 2), observations with missing covariates were excluded.

Eligible Sample Characteristics:

© 2025 Kotb et al. *JAMA Network Open.*

eTable 11. Eligible Sample Characteristics

	(1)
	Share % (95% CI)
Demographic Characteristics	
Age Under 65 (Qualified through a disability)	34.4 (32.4, 36.4)
Not married	74.9 (73.0, 76.8)
High School Degree or Lower	67.1 (64.8, 69.4)
Female	61.4 (59.4, 63.4)
Limited English Proficiency	16.4 (14.1, 18.7)
Race and Ethnicity	
White	69.0 (66.0, 72.0)
Black or African American)	25.4 (22.5, 28.3)
Asian	3.8 (2.7, 5.0)
AIAN and NHPI	2.4 (1.2, 3.6)
Hispanic Ethnicity	20.0 (17.5, 22.5)
Economic Characteristics	
Income under 100% FPL	63.7 (60.9, 66.4)
Assets under $3,000	87.9 (85.9, 89.8)
Area Deprivation Index – Top Quartile	37.4 (33.5, 41.4)
Urban residence	77.3 (74.0, 80.7)
Food insecure	32.3 (30.5, 34.2)
Health and Daily Activities	
Moderately Severe or Severe Depression	28.5 (26.7, 30.2)
Vision Problems	13.1 (12.0, 14.1)
Hard of Hearing	15.9 (14.7, 17.1)
Difficulty in Any Activity of Daily Life	40.1 (38.5, 41.7)
Difficulty in Any Instrumental Activity of Daily Life	70.3 (68.4, 72.2)
Person-Years	35,587,360

Notes: Race is self-reported and survey respondents could choose more than one race. We do not report AIAN and NHPI separately due to small samples and CMS restrictions on minimum required cell sizes for reporting. NHPI indicates Native Hawaiian or Pacific Islander race; AIAN, American Indian or Alaska Native race; ADL indicates activity of daily living; and IADL, instrumental activity of daily living.

© 2025 Kotb et al. *JAMA Network Open.*

eAppendix 5. Robustness Checks

eFigure 6. Robustness of Take-Up to Income and Asset Definition

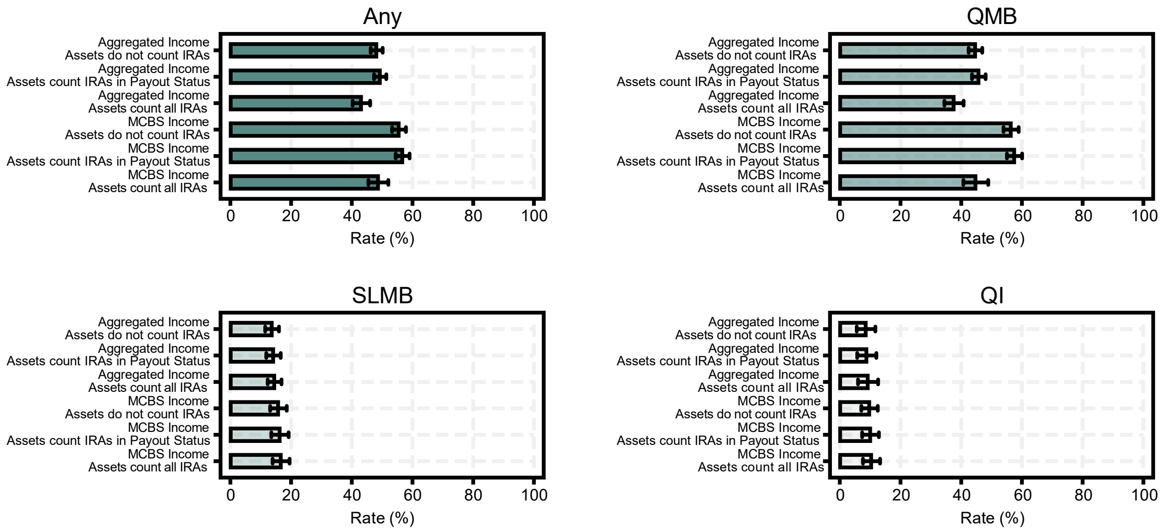

Notes: The graph presents take-up rates using the different income and asset definitions described above, by MSP subprogram. QMB refers to the Qualified Medicare Beneficiary program, SLMB refers to the Specified Low-Income Medicare Beneficiary program, and QI refers to the Qualifying Individual program. Enrollees can shift from one program to the other mid-year. As such, we consider an individual to be enrolled in a specific MSP subprogram in a year if the modal number of enrolled months in that year points to this program.

© 2025 Kotb et al. *JAMA Network Open.*

eAppendix 6. Characteristics of the Enrolled and Unenrolled Eligibles

eTable 12. Differences in Characteristics of the Enrolled and Unenrolled Eligibles

	(1)
	Difference p.p. (95% CI)
Demographic Characteristics	
Age Under 65 (Qualified through a disability)	12.8 (9.1, 16.6)
Not married	16.3 (12.6, 20.0)
High School Degree or Lower	9.1 (4.5, 13.7)
Female	2.7 (-1.1, 16.5)
Limited English Proficiency	11.4 (8.8, 13.9)
Race and Ethnicity	
Asian	3.3 (1.8, 4.8)
AIAN and NHPI	0.6 (-0.7, 1.9)
Black or African American	2.1 (-1.9, 6.1)
Hispanic	9.6 (6.8, 12.5)
White	-5.6 (-9.4, -1.8)
Economic Characteristics	
Income under 100% FPL	30.0 (25.4, 34.6)
Assets under $3,000	16.4 (13.2, 19.6)
Area Deprivation Index: Top Quartile	7.0 (2.2, 11.7)
Urban residence	3.7 (0.5, 6.9)
Food insecure	10.2 (6.8, 13.5)
Health and Daily Activities	
Moderately Severe or Severe Depression	9.3 (6.0, 12.7)
Vision Problems	4.9 (2.8, 7.0)
Hard of Hearing	1.6 (-1.3, 4.4)
Difficulty with any ADL	10.4 (7.2, 13.6)
Difficulty with any IADL	11.1 (7.6, 14.6)

Notes: Race is self-reported and survey respondents could choose more than one race. We do not report AIAN and NHPI separately due to small samples and CMS restrictions on minimum required cell sizes for reporting. NHPI indicates Native Hawaiian or Pacific Islander race; AIAN, American Indian or Alaska Native race; ADL, activity of daily living; and IADL, instrumental activity of daily living.

© 2025 Kotb et al. *JAMA Network Open.*

eTable 13. Differences in Assets among the Enrolled and Unenrolled Eligibles

	(1)
	Difference $1,000s (95% CI)
Liquid Assets	
Cash	-5.8 (-7.9, - 3.7)
Illiquid Assets	
Stocks, bonds, mutual funds	-198.8 (-403.7, 6.2)
Property	-352.7 (-646.1, -59.3)
Retirement funds	-181.8 (-390.3, 26.6)

Notes: Retirement accounts include those that are in payout status. Property excludes primary residence value, which does not count against MSP eligibility.

© 2025 Kotb et al. *JAMA Network Open.*

eFigure 7. Characteristics of the Enrolled and Unenrolled Eligibles, With State Fixed Effects

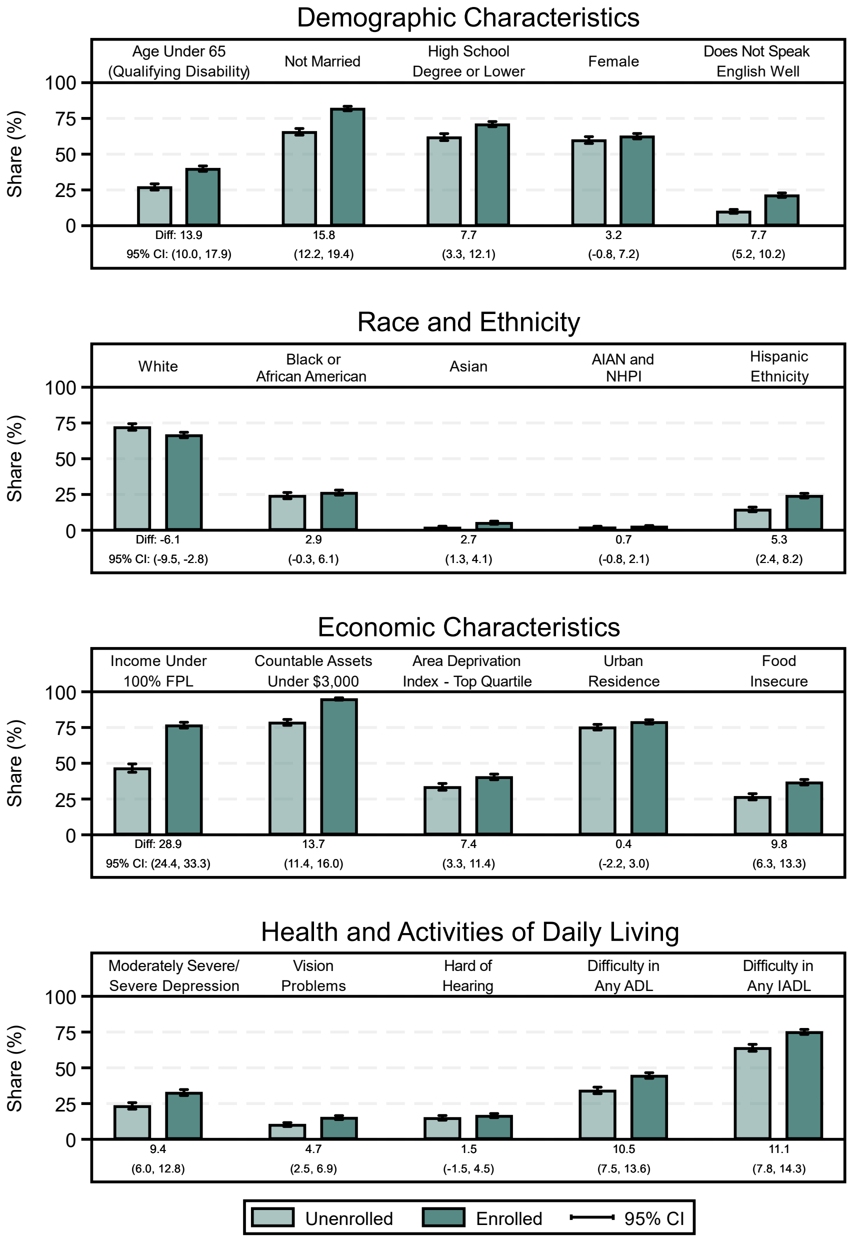

Notes: The graph presents the share of unenrolled eligible and enrolled individuals who exhibit a particular characteristic. Race is self-reported and survey respondents could choose more than one race. We do not report NHPI and AIAN races separately due to small samples and CMS restrictions on minimum required cell sizes for reporting. NHPI indicates Native Hawaiian or Pacific Islander race; AIAN American Indian or Alaska Native race; ADL, activity of daily living; and IADL, instrumental activity of daily living.

© 2025 Kotb et al. *JAMA Network Open.*

eFigure 8. Comparison of Assets Among the Enrolled and Unenrolled Eligibles, With State Fixed Effects

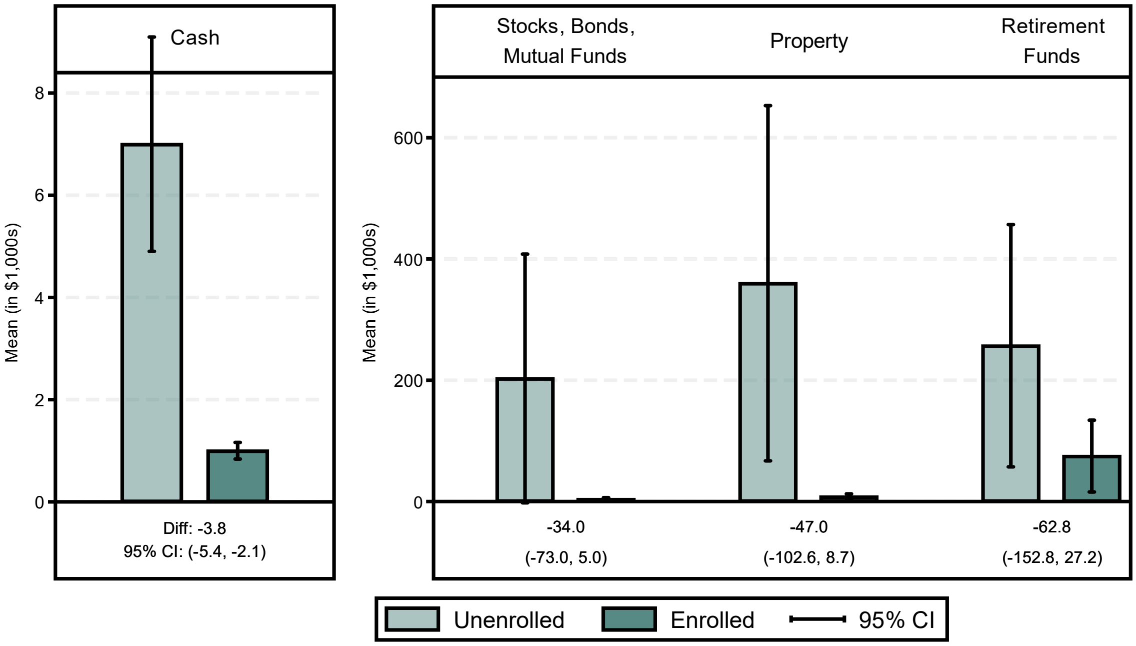

Notes: The graph presents assets by type among the unenrolled eligible and the enrolled populations. Retirement accounts include those that are in payout status. The "Property" category excludes primary residence value, which does not count against MSP eligibility. The differences reported control for state dummy variables.

© 2025 Kotb et al. *JAMA Network Open.*

eReferences.

1. NCOA. Medicare Savings Programs (MSPs): Eligibility and Coverage (2018). March 2018. Accessed October 22, 2024. https://www.benavest.com/wp-content/uploads/2019/01/Summary-of-Medicare-Savings-Program-Eligibility-and-Coverage-by-NCOA.pdf

2. NCOA. Medicare Savings Programs (MSPs): Eligibility and Coverage (2019). February 2019. Accessed October 22, 2024. https://lists.ncoa.org/mippa/cache/10522477/2.pdf

3. NCOA. Medicare Savings Programs (MSPs): Eligibility and Coverage (2020). February 2020. Accessed September 13, 2024. https://lists.ncoa.org/bec/cache/13309073/2.pdf

4. Louisiana Department of Health. Louisiana Medicaid Eligibility Manual: Medicare Savings Program Resource Limits: Published online August 25, 2020. Accessed July 17, 2024. https://ldh.la.gov/assets/medicaid/MedicaidEligibilityPolicy/Z-2200.pdf

5. Southern Maine Agency on Aging. Financial Guidelines to Commonly Used Programs. Published online March 1, 2020. https://www.smaaa.org/documents/cs/medicare/financial_income_guidelines.pdf

6. Oliver T, Zhu W, Lakhmani EW, Wysock A. *Medicare Savings Program Enrollment Increases When States Expand Financial Eligibility Criteria*. AARP; 2023. doi:10.26419/ppi.00210.001

7. Illinois Department of Healthcare and Family Services. Illinois State Plan Amendment. Published online April 24, 2020. Accessed July 17, 2024. https://www.medicaid.gov/State-resource-center/Medicaid-State-Plan-Amendments/Downloads/IL/IL-20-0004.pdf

8. Maine Department of Health and Human Services. 2020 MaineCare Eligibility Guidelines. 2020. Accessed November 27, 2024. https://www.maine.gov/dhhs/documents/2020-MaineCare-Eligibility-Guidelines.pdf

9. Maine Department of Health and Human Services. 2018 MaineCare Eligibility Guidelines. 2019. Accessed November 27, 2024. https://mainefamilyplanning.org/wp-content/uploads/MaineCare-Eligibility-Guidelines-100319.pdf

10. SSA. Medicare Savings Programs Income Limits. November 13, 2023. Accessed October 28, 2024. https://secure.ssa.gov/poms.nsf/links/0600815023

11. Centers for Medicare and Medicaid Services. 2018 Medicare Current Beneficiary Survey Data User's Guide: Survey File. Published online 2018. https://www.cms.gov/research-statistics-data-and-systemsresearchmcbscodebooks/2018-mcbs-survey-file

© 2025 Kotb et al. *JAMA Network Open.*
