# Supplementary material for: Medicare Savings Program Take-Up Estimates and Profile of Enrolled and Unenrolled Individuals
Source: JAMA Netw Open. 2025 Oct 3;8(10):e2535408. doi: 10.1001/jamanetworkopen.2025.35408 (PMC12495494; doi:10.1001/jamanetworkopen.2025.35408)
Supplement: Supplement 2. — Data Sharing Statement [file jamanetwopen-e2535408-s002.pdf]

Data Sharing Statement

Kotb. Medicare Savings Program Take-Up Estimates and Profile of Enrolled and Unenrolled Individuals. *JAMA Netw Open*. Published October 03, 2025.
doi:10.1001/jamanetworkopen.2025.35408

Data
Data available: No

Additional Information
Explanation for why data not available: Data access is governed by a DUA that prohibits public redistribution.
